# Screening of methicillin-resistant *Staphylococcus aureus* nasal colonization among elective surgery patients in referral hospital in Indonesia

**DOI:** 10.1186/s13104-018-3150-y

**Published:** 2018-01-22

**Authors:** Erni J. Nelwan, Robert Sinto, Decy Subekti, Randy Adiwinata, Lia Waslia, Tonny Loho, Dodi Safari, Djoko Widodo

**Affiliations:** 1 Division of Tropical and Infectious Diseases, Department of Internal Medicine, Faculty of Medicine Universitas Indonesia, Dr. Cipto Mangunkusumo General Hospital, Jl. Salemba Raya 6, Jakarta, 10430 Indonesia; 2 Eijkman Oxford Clinical Reseach Unit, Jl. Diponegoro No. 69, Jakarta, 10430 Indonesia; 3 Department of Clinical Pathology, Faculty of Medicine Universitas Indonesia, Dr. Cipto Mangunkusumo General Hospital, Jl. Salemba Raya 6, Jakarta, 10430 Indonesia; 4 Eijkman Institute for Molecular Biology, Jl. Diponegoro No. 69, Jakarta, 10430 Indonesia

**Keywords:** Methicillin-resistant *Staphylococcus aureus*, Nasal colonization, Pre-operative

## Abstract

**Objective:**

Methicillin-resistant *Staphylococcus aureus* (MRSA) colonization is associated with serious surgical site infection in high-risk patients. High prevalence of MRSA colonization was reported in many settings, nonetheless local data is required. The purpose of this study is to identify the prevalence and risk factor of MRSA nasal carriage in adult patients in National Referral Hospital in Indonesia before underwent elective surgical procedure.

**Results:**

From 384 patients, 16.9% patients of them had undergone orthopaedic surgery, 51.3% had received antibiotics within the previous 3-month and 41.1% patients had history of hospitalization within the previous 1 year. Total of 21.6% patients were on invasive devices for at least 48 h before the operation; 24.2% had an open wound; 19.3% patients were referred from other hospital/ward. Of these patients, solid tumor without metastasis was the most common factor identified by the Charlson index (38.3%). Nasal colonization of Gram-positive bacteria was detected in 76.8%; *S. aureus* in 15.6% of patients (n = 60). MRSA was identified in three isolates (0.8%) by both culture and polymerase chain reaction (PCR) tests. Due to low prevalence of MRSA nasal carriage, this finding supports the recommendation to not routinely apply mupirocin for nasal decolonization on patient planned for surgery in Indonesia.

**Electronic supplementary material:**

The online version of this article (10.1186/s13104-018-3150-y) contains supplementary material, which is available to authorized users.

## Introduction

Surgical site infection (SSI) remains a major contributor of healthcare associated infection (HAI). Based on Centers for Disease Control and Prevention (CDC) prevalence survey in 2011, there were 157,500 SSI cases among all infections occurred in hospitals [1]. SSI was associated with substantial increased of postoperative hospital stay, rates of hospital readmission, functional disability, hospital cost, and mortality rate [2]. *Staphylococcus aureus* remains the leading cause of SSI, with half of the *S. aureus* were found resistant to methicillin [3, 4]. SSI caused by *Methicillin*-*Resistant S. aureus* (MRSA) are increasing in proportion and associates with more devastating outcome [2, 5]. Numerous studies found that patients with nasal colonization of MRSA were associated with higher risk of SSI [6–8]. Routine decolonization of MRSA with chlorhexidine bathing and mupirocin nares application before surgery is becoming an interesting strategy option to reduce number of SSI, especially in hospital with high rate of MRSA colonization [6, 9, 10]. However, this strategy is not free of consequence. The emergence of chlorhexidine-resistant bacteria and mupirocin resistance are two concerns raised with the wide spread application of this strategy. Active screening followed by selective decolonization is another strategy but with associated with relatively higher cost due to additional diagnostic expense [11]. We aimed to investigate the prevalence of MRSA nasal carriage, to determine suitable MRSA decolonization strategy for surgical patients in Indonesia.

## Main text

### Study design and population

The cross sectional study was conducted in Cipto Mangunkusumo Hospital, Jakarta, Indonesia, between April and September 2015 which was established as a referral hospital for all over the country (260 million inhabitant). Total capacity for inpatient is 900 beds; with approximately 36,000 inpatients from all wards per year and more than 40,000 surgeries per year. To collect data on MRSA colonization among elective surgical patients, we screened all adult patients admitted to the hospital and assigned for an elective surgery. Two hours prior to screening procedure, the list of all adult patients that were planned for elective surgery in the next 24 h were received. Those who met with the inclusion criteria: aged ≥ 18 years old, assigned for an elective surgery in the next 24 h, and willing to participate in the study were included consecutively. Patients with active MRSA infection, contraindication for nasal manipulation, and underwent obstetric-gynecology procedure were excluded in this study. Written informed consent was asked before specimens from nasal swab were taken.

### Specimen collection

Nasal swabs were collected using a sterile dry cotton swab from all eligible patients approximately 12 h before the surgery. Swabs were placed into Stuart Transport Medium and was inoculated onto sheep blood agar plate and mannitol salt agar plate (Oxoid, Hamshire, UK). The culture plates were examined after 24–48 h of incubation at 35 °C. The presumptive *S. aureus* bacteria were than confirmed by catalase and oxidase tests and stored in Tryptone Soya Broth-Glycerol for further analysis [12]. Other bacterial isolates were identified using a standard protocol in the Department of Clinical Pathology, Cipto Mangunkusumo General Hospital, Jakarta.

### Antimicrobial susceptibility testing

Antibiotic susceptibility testing was performed by the disk diffusion method using guidelines established by the Clinical and Laboratory Standards Institute (CLSI) [13]. A total of seven selected antibiotic disks (Oxoid, Hamshire, UK) were used in the test. Those were chloramphenicol (30 μg), erythromycin (15 μg), tetracycline (30 μg), cefoxitin (30 μg), oxacillin (1 μg), gentamicin (10 μg) and trimethoprim-sulfamethoxazole (25 μg). CLSI guidelines were used for interpretating the zones of inhibition [13]. The organism use for quality control was *S. aureus* ATCC 25923.

### Detection of *mecA* and *nuc* genes

A few colonies was suspended in TE buffer (10 mM Tris–HCl, 1 mM Na_2_EDTA, pH 8.0) solution and heated at 100 °C for 10 min and instantly frozen at − 20 °C for 5 min. The lysates were centrifuged at 1000×*g* for 10 min. Polymerase chain reaction (PCR) targeting staphylococcal nuclease (*nuc*) and methicillin-resistance (*mecA*) genes for detection of *S. aureus* and MRSA isolates were performed as described previously [12, 14, 15]. In short, the reaction mixture contained GoTaq Green Master Mix (Promega), Primers for amplification of *nuc genes*: 5′-TCAGCAAATGCATCACAAACAG-3′ and 5′-CGTAAATGCACTTGCTTCAGG-3′ and primers for amplification of *mecA gene*: 5′-GGGATCATAGCGTCATTATTC-3′ and 5′-AACGATTGTGACACGATAGCC-3′ at 10 μM concentration, and 1 μl of DNA template. The presence of 255 bp amplicon for *nuc* and 527 bp amplicon for *mecA* respectively were detected by electrophoresis on 1.5% agarose gel and visualized with SYBR safe DNA gel stain (Invitrogen, USA).

### Statistical analysis

All data was processed using Statistical Program for Social Sciences (Version 20; SPSS Inc., Chicago, IL, USA) and results will be presented descriptively.

## Results

**Fig. 1:**
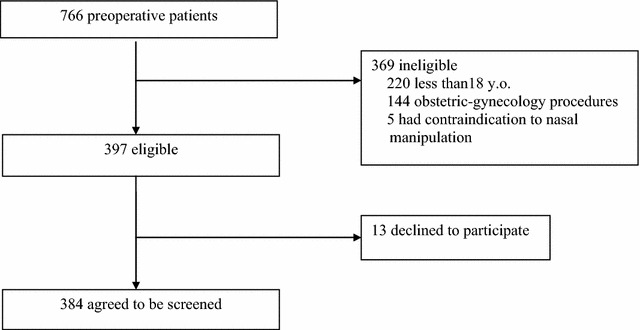
The flowchart of patients selection

Between April and September 2015, a total of 766 patients undergoing various surgeries were screened and 397 patients were eligible for the study. Among them, 384 patients gave written consent to participate in the study (see Fig. 1). The median age of study population was 46 (IQR:25.0) years old and 41.9% were male. The median length of stay the patients before operation were 2 (IQR:8.0) days. Among 384 patients, 65 (16.9%) underwent orthopedic surgery, 60 (15.6%) for digestive surgery, 60 (15.6%) for ear-nose-throat surgery and 58 (15.1%) for oncology surgery (Table 1).

**Table 1:** Characteristics of participant

	N (%)
Male	161 (41.9)
Age (years old), median (interquartile)	46 (25.0)
Type of operation	
Orthopedics	65 (16.9)
Digestive	60 (15.6)
Ear-nose-throat	60 (15.6)
Oncology	58 (15.1)
Vascular	38 (9.9)
Cardiology	29 (7.6)
Neurology	28 (7.3)
Oral	27 (7.0)
Plastic-reconstruction	9 (2.3)
Urology	7 (1.8)
Comorbidities	
Tumor without metastases	147 (8.3)
Congestive heart failure	29 (7.6)
Diabetes with end organ damage	28 (7.3)
Charlson comorbidity index, median (IQR)	2 (2)

### MRSA risk factor and patient’s comorbidities

The median score of 2 (IQR: 2) from Charlson comorbidity index to determine the risk of MRSA colonization (see Additional file 1). In addition to that, other risk factors such as history of antibiotics usage in the past 3 months (51.3%), history of hospitalization in the last 12 months (41.1%) were also identified among this group of patients. More than 20% of patients had open wound and were on invasive devices for 48 h prior to the screening; and 19.3% patient were referred from other hospital or transferred from other wards. Neither history of past colonization and infection of MRSA nor history of living in nursing home were identified.

### *Staphylococcus aureus* and MRSA nasal colonization

The vast majority of nasal swab cultures results were Gram-positive bacteria (76.5%). A total of 168 patients (43.8%) were colonized with *Staphylococcus epidermidis* and 57 patients (14.8%) had positive nasal swabs for *Methicillin*-*Sensitive Staphylococcus aureus* (MSSA). (Table 2.) Three isolates that showed MRSA were determined by cefoxitin diffusion test and PCR therefore given the prevalence rate of 0.8% (Table 2).

**Table 2:** Microbiological culture findings

Bacteria	N (%)
*Methicillin*-*sensitive Staphylococcus aureus*	57 (14.8)
*Methicillin*-*resistant Staphylococcus aureus*	3 (0.8)
*Staphylococcus epidermidis*	168 (43.8)
*Staphylococcus saprophyticus*	57 (14.8)
*Klebsiella pneumonia*	27 (7.0)
*Bacillus* sp.	26 (6.8)
*Enterobacter enterogenes*	19 (4.9)
*Escherichia coli*	9 (2.3)
Others*	18 (4.8)

* Alpha-hemolytic *Streptococcus* sp. (1.8), *Acinetobacter baumannii* (0.5), Non-hemolytic *Streptococcus* sp. (0.5), *Pseudomonas aeruginosa* (0.5), *Streptococcus viridans* (0.3), *Acinetobacter lwoffii* (0.3), *Citrobacter freundii* (0.3), *Enterobacter faecalis* (0.3)

### MRSA positive patients

The colonization of MRSA was found in three patients planned for orthopaedic, vascular and digestive surgery. These patients were hospitalized in minimum of 1–5 days before cultures were taken. The Charlson comorbidity index was two for two patients and six for one patient.

## Discussion

During study period, we found a very low prevalence (0.8%) of MRSA nasal carriage by culture and PCR among preoperative patients compared to report from other countries. One hospital in Singapore found the prevalence of MRSA colonization was 10.6% [16]. While Hadley, et al. reported prevalence of MRSA colonization anterior nasal was 3.5% among patients underwent total joint replacement in hospital in United States [17]. Prevalence of 4.25% was reported in retrospective study among cardiothoracic and neurological surgical patients in United States [18]. A recent study by Santosaningsih, et al. found that MRSA carriage rate was 4.3% among surgical patients in three academics hospitals in Indonesia. In the study, the screening was performed at the time of discharge and patients that discharged within 48 h of admission were excluded. Subgroup analysis of three hospitals that participated in this study revealed a significant variation number of MRSA carriage, i.e. 8.0% in Malang-East Java, 5.9% in Semarang-Central Java and 0.4% in Denpasar-Island of Bali [19]. More recent publication from Kuntaman found MRSA prevalence of 3.9% from nasal swab among patients on admission to medical wards in Surabaya [20]. Difference in prevalence rate between each hospital, reflected the need of local data to assess the needs of routine or selective decolonization protocol in preoperative patients.

The low number of MRSA colonization in our study may be influenced by short duration of hospitalization (median = 2 days) before the specimens were obtained. This may reflect the low burden of community-acquired MRSA in Indonesia [21]. In addition, no patient had history of MRSA infection and lived in nursing home, which in many studies reported as important risk factor of MRSA colonization [16, 22, 23].

Our subjects had well-known significant independent risk factors of MRSA such as the history of antibiotics usage, invasive medical instrumentation, history of recent hospitalization, open wound, and also mixed cases of medical comorbidities which assessed with Charlson comorbidity index. Some of comorbidities that related with MRSA infection were diabetes, chronic kidney disease, skin infections, pulmonary disease, congestive heart failure, and immunosuppression [24, 25].

Low prevalence of MRSA colonization in this study supports the previous study of low MRSA colonization burden in Indonesia [19, 20]. Therefore, routine decolonization strategy may not be appropriate to be implemented in Indonesia. Routine screening to identify MRSA colonization by culture are not only cost burdening for developing countries, but also time consuming [26, 27]. While PCR can give rapid result for screening, it is not readily available in all hospitals. Routine decolonization with mupirocin may more cost effective however it may raise the possibility of resistance and lead to treatment failure [4, 5]. Nowadays, there were increasing trend of mupirocin resistance among MRSA, some of them were caused by extended and excessive use of mupirocin ointment [28, 29]. Selective swabbing and decolonization for high risk patients may be more appropriate for limited resources countries [26].

To summarize, a low prevalence of MRSA nasal carriage was found among patients performed elective surgery in the national referral hospital in Indonesia. This finding may supports the recommendation to not routinely apply mupirocin for nasal decolonization on patient plan for surgery in Indonesia.

### Limitations

Our study was limited to only one site swabbing for the MRSA colony. Therefore, We might miss the MRSA colony in other areas such as axilla, throat, perineum, and anal [19], although anterior nasal is the highest site of colonization [3]. Another limitation is the one-center study that threatened the external validity to be generalized in Indonesia. Further similar prevalence study should be done across all hospitals in Indonesia, to accurately describe the burden of MRSA colonization among preoperative patients.

## Additional file

**Additional file 1.** Comorbidities of the participants

## Abbreviations

MRSA: methicillin resistant *Staphylococcus aureus*
MSSA: methicillin sensitive *Staphylococcus aureus*
HAI: healthcare associated infection
CDC: Centers for Disease Control and Prevention
PCR: polymerase chain reaction
SBA: sheep blood agar
MSA: mannitol salt agar
CLSI: Clinical and Laboratory Standards Institute
TE: Tris–EDTA
