# Supplementary material for: Screening of methicillin-resistant Staphylococcus aureus nasal colonization among elective surgery patients in referral hospital in Indonesia
Source: BMC Res Notes. 2018 Jan 22;11:56. doi: 10.1186/s13104-018-3150-y (PMC5778668; doi:10.1186/s13104-018-3150-y)
Supplement: Supplementary file 1 — Additional file 1. Comorbidities of the participants [file 13104_2018_3150_MOESM1_ESM.docx]

Additional file 1. Comorbidities of the participants

|  | N (%) |
| --- | --- |
| *Charlson comorbidity index*  Myocardial infarction | 25 (6.5) |
| Congestive heart failure | 29 (7.6) |
| Peripheral artery disease | 6 (1.6) |
| Cerebrovascular disease | 14 (3.6) |
| Dementia | 1 (0.3) |
| Chronic pulmonary disease | 1 (0.3) |
| Connective tissue disease | 1 (0.3) |
| Peptic ulcer disease | 1 (0.3) |
| Diabetes |  |
| Yes, with end-organ damage | 28 (7.3) |
| Yes, without end-organ damage | 12 (3.1) |
| Mild liver disease | 9 (2.3) |
| Moderate or severe renal disease | 19 (4.9) |
| Hemodialysis | 14 (3.6) |
| Hemiplegia | 8 (2.1) |
| Leukemia | 0 (0) |
| Lymphoma | 3 (0.8) |
| Tumor without metastases | 147 (38.3) |
| Metastatic solid tumor | 10 (2.6) |
| AIDS | 0 (0) |
